# Measurement of the retinal venous pressure with a new instrument in patients with primary open angle glaucoma

**DOI:** 10.1007/s00417-023-06326-4

**Published:** 2024-01-12

**Authors:** Richard Stodtmeister, Aline Menzel, Anna Klimova, Robert Herber, Karin. R. Pillunat, Lutz E. Pillunat

**Affiliations:** 1 Department of Ophthalmology, Univ. Hospital Carl Gustav Carus, TU Dresden, Fetscherstrasse 74, 01307 Dresden, Germany; 2 National Center for Tumor Diseases (NCT/UCC), German Cancer Research Center (DKFZ), Faculty of Medicine and University Hospital Carl Gustav Carus, Technische Universität Dresden, Helmholtz-Zentrum Dresden-Rossendorf (HZDR), 01307 Dresden, Germany

**Keywords:** Retinal veins, Venous pressure, Ocular circulation, Intraocular pressure, Ophthalmodynamometry, Data accuracy

## Abstract

**Purpose:**

To compare the results of retinal venous pressure (RVP) measurement performed with contact lens dynamometry (CLD) and with the new IOPstim.

**Methods:**

In this cross-sectional study, we included 36 patients with primary open angle glaucoma with a median age (Q25; Q75) of 74 (64; 77) years (m/f = 18/18), baseline intraocular pressure (IOP): 13.9 (12.2; 15.1) mmHg. Median mean defect: − 5.8 (− 11.9; − 2.6) db. Principle of the IOPstim: an empty balloon with a diameter of 8 mm is positioned on the eye, laterally of the limbus. Under observation of the central retinal vein (CRV), the examiner inflates the balloon. As soon as the CRV starts pulsation, the inflation is stopped and the IOP is measured, equaling the RVP at this moment. In the CLD, the pulsation of the CRV is observed with a contact lens. The RVP is calculated from the attachment force applied when pulsation appears.

**Course of examinations:**

Three single measurements of RVP in quick succession with both methods. The sequence of the two methods was randomized. The means of the three RVP measurements were compared.

**Results:**

Pressures in mmHg. RVP: IOPstim: 19.4 ± 5.4 (mean ± SD), CLD: 20.3 ± 5.9. Range of three single measurements: IOPstim: 2.9 ± 1.5, CLD: 2.2 ± 1.1. The differences were RVP_IOPstim_ − RVP_CLD_ =  − 0.94 ± 1.15, and approximately normally distributed. Bland–Altman analysis: only one data point was 0.5 mmHg higher than the upper line of agreement. The confidence interval of this line was 0.65 mmHg. Concordance correlation coefficient according to Lin (CCC): 0.96. Intraclass correlation coefficient: both methods, 0.94.

**Conclusion:**

In both methods, the range of the single measurements may be taken as a sign of good reliability, the CCC of 0.96 as a sign of a very good agreement. At the mean, the IOPstim RVP values were 1 mmHg lower than those obtained with the CLD. This difference may be due to the different directions of the prevailing force vectors induced by the instruments. The IOPstim seems applicable in glaucoma diagnostics.

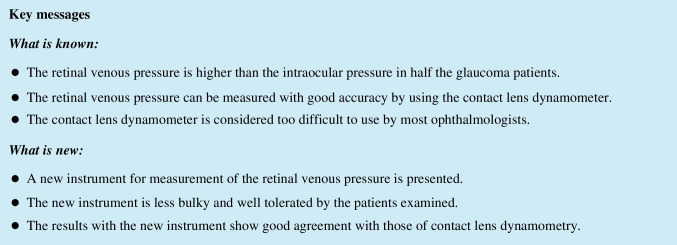

## Introduction

**Fig. 1:**
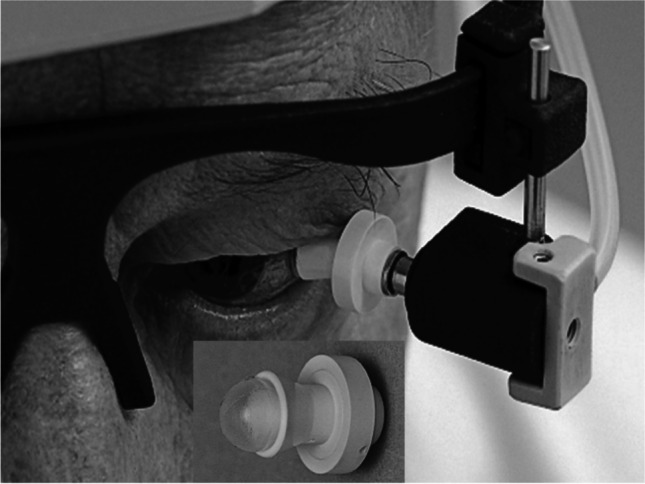
The IOPstim positioned on the surface of the eye, the balloon is deflated. Inset: balloon moderately inflated

The retinal venous pressure (RVP) is a better predictor of future optic disc excavation than the intraocular pressure (IOP) [1–3] in patients with primary open angle glaucoma (POAG). Until now, this has been measured with the contact lens dynamometer (CLD) [4–6]. This instrument, however, did not find widespread use because of its bulky shape and its sensitivity to improper handling compared to a common 3-mirror contact lens. Therefore, a new instrument (Fig. 1) was developed [7] from which reliable results could be obtained and which was well accepted by healthy subjects as well as the examiner [8]. This first study had to be performed under the use of the Valsalva maneuvre (VM) because healthy subjects show a spontaneous pulsation of the CRV in the vast majority of cases [9]. By applying the VM, a spontaneous pulsation vanishes and can be elicited again by an artificial increase of the IOP [10]. Median RVP values increased by 13 mmHg (1.5times) when obtained with the CLD. The explanation was the main force vector of the CLD is in the direction of the apex of the orbit and thereby directly in opposition to the force of the retained blood in the CRV. In *glaucoma patients*, the CRV does not pulsate in a high percentage of cases [11] and, therefore, the measurement of the RVP is possible without the VM. In the current study, we measured the RVP in patients with POAG without spontaneous pulsation and tested the hypothesis that the RVP values do not differ when obtained by either of the methods.

## Methods

### Subjects and procedure

In this cross-sectional study, the measurements were performed in 36 patients with POAG (Table 1) who provided informed consent before enrolment. The inclusion criteria were as follows: POAG according to the definition of the European glaucoma society [12], a pupil diameter in mydriasis of ≥ 6 mm, no pulsation of the CRV. The exclusion criteria were as follows: incisional intraocular surgery (except in cases of uneventful phacoemulsification more than 6 months prior); refractive corneal surgery; right-sided heart failure; inflammation of the outer eye or within the eye; status after retinal detachment; myopia >|4,9| dioptres (spherical equivalent); and allergy to the applied eye solutions. The more advanced eye was included according to the criteria of mean defect, excavation, and IOP.

**Table 1:** Description of the patients. *BMI* body mass index, *IOP* intraocular pressure, *MAP* mean arterial pressure

Demographics of patients		
*N* = 36, m/f = 18/18		
	Mean	*s*
Age, years	69	13
BMI, kg/m^2^	25.5	3.9
Baseline IOP, mmHg	13.8	3.3
IOP, mmHg, mydriasis	13.6	3.3
MAP before IOPstim, mmHg	106	11
MAP before CLD, mmHg	108	12
Mean defect, db	− 10.3	7.9

Two methods of RVP measurement were used: the new IOPstim (Imedos Health GmbH, Jena, Germany) and the CLD device (same manufacturer). The principle of the IOPstim is that an empty balloon with a diameter of 8 mm (more precisely a pelotte) is positioned on the eye, laterally of the limbus (Fig. 1). Under observation of the central retinal vein (CRV) and their major branches on or near the optic disc, the examiner inflates the balloon. As soon as the CRV pulsates, the inflation is stopped and the IOP is measured by rebound tonometry (RT; iCare, Tiolat Oy, Vantaa, Finland) which equals the RVP at this moment.

The other method was contact lens dynamometry (CLD) (Imedos Health GmbH, Jena, Germany; former manufacturer: Meditron, Voelklingen, Germany). This instrument consists of a commercially available Goldmann 3-mirror contact lens (Haag-Streit, Koeniz, Switzerland), which is connected to a metal ring by strain gauges. The signal is transmitted by a flexible wire to the electronic central unit, which shows the induced pressure increase in mmHg. A pulsation is defined as the change of appearance of a retinal venous vessel on or near the optic disc which in turn is in phase with the arterial pulse. More details concerning the method have been previously published [13, 14].

The course of the examination was as follows: IOP measurement followed by installation of tropicamide eye solution 5 mg/ml (Mydrum, Bausch and Lomb, Berlin), IOP measurement in mydriasis, semiautomatic systemic blood pressure measurement (Omron 5 Professional, Omron, Kyoto, Japan), and three measurements of RVP by either the IOPstim or CLD in quick succession. Afterwards, the same procedure was performed with the remaining method. The sequence of the measurement methods had been randomized beforehand by an urn model without layback, in blocks of ten. Finally, the IOP was measured. All IOP measurements were performed with RT. The cooperation of the patients was rated using four classes and the agreeability was assessed by a five-stage classification system as previously used and described [8]. According to the manufacturer, the cost of each of the two instruments is in the order of €9500.

The distribution of RVP values was assessed using P-P diagrams and tested by Kolmogorov–Smirnov test, by Lillefors test and by Shapiro-Wilks test. The agreement analysis was carried out using the Bland Altman method [15], as recommended by McAlinden et al. [16], among others. To obtain an additional insight, the concordance correlation coefficient according to Lin was calculated, as suggested by Koch and Spoerl [17].

In case of a normal distribution, the data were expressed as mean ± s. A *p*-value lower than 0.05 was considered statistically significant.

## Results

The demographics of the patients is shown in Table 1.

The results of the RVP measurements are shown in Table 2.

**Table 2:** The results obtained with IOPstim and CLD. *BAIOP* baseline IOP, *RVP* retinal venous pressure, *SM* single measurement, *ICC* intraclass correlation coefficient, *CCC* concordance correlation coefficient according to Lin [17], *CI* confidence interval

Results, *N* = 36						
	IOPstim			CLD		
	Mean	*s*	95% CI	Mean	*s*	95% CI
BAIOP	13.5	3.5	12.3–14.7	13.6	3.2	12.2–14.4
RVP	19.4	5.4	17.6–21.2	20.3	5.9	18.4–22.3
ICC in 3 SM	0.94			0.94		
CCC	0.96 (CI: 0.93–0.98)					

**Fig. 2:**
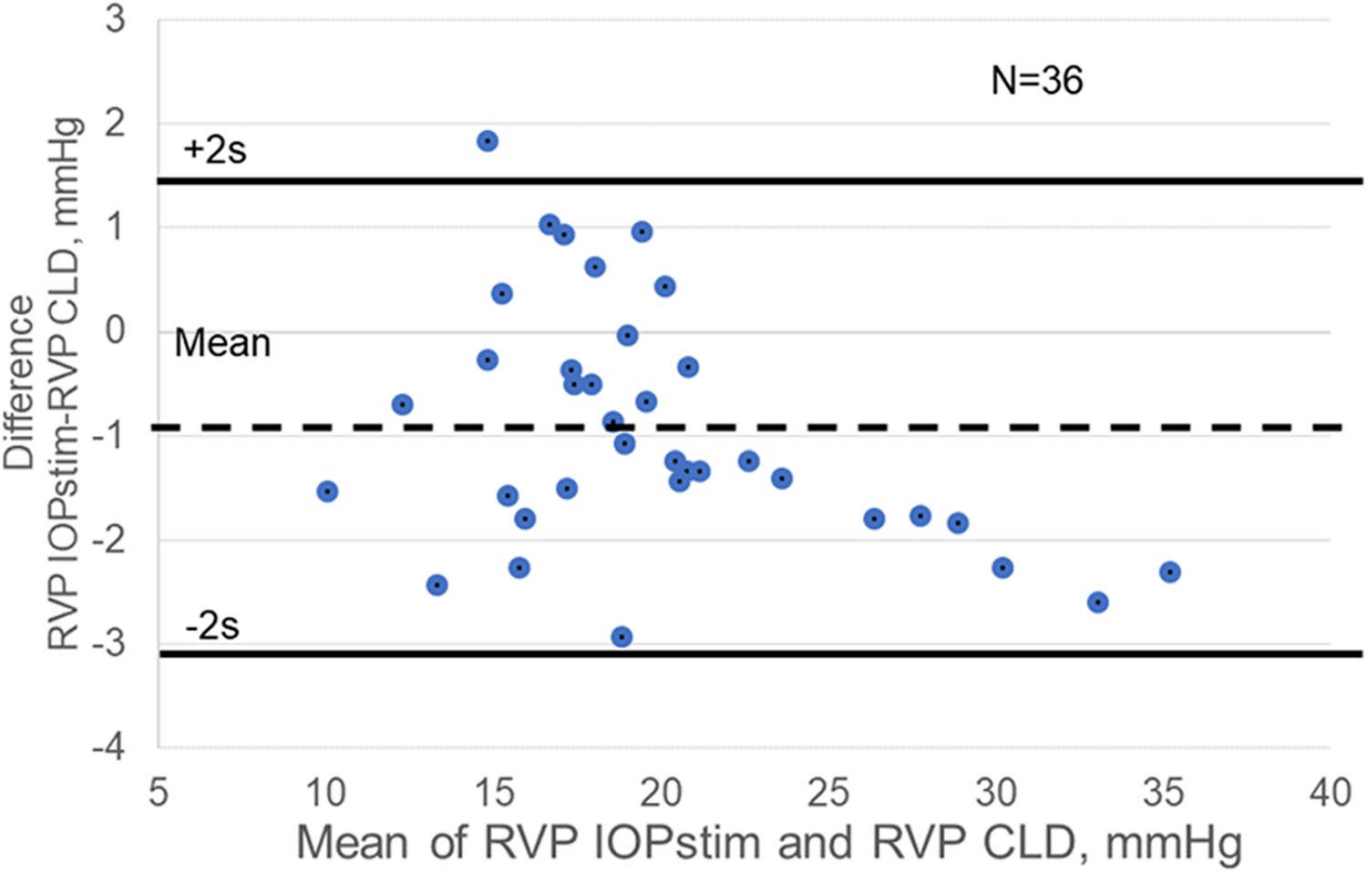
Bland-Altman diagram showing the RVP values obtained by the two methods (IOPstim and CLD). Abbreviations: RVP, retinal venous pressure; CLD, contact lens dynamometry. Abscissa: mean of RVP measured by IOPstim and RVP measured by CLD. Ordinate: difference—RVP measured by IOPstim minus RVP measured by CLD

Assessed according to the P-P diagrams, the differences of the RVP values (RVP_IOPstim_ – RVP_CLD_) showed no significant deviation from the normal distribution. The test results for normal distribution were as follows: Kolmogorov–Smirnov test—*d* = 0.12973, *p* > 0.20; Lillefors test: *p* < 0.15; Shapiro-Wilks test—*W* = 0.96645, *p* = 0.33659. Thus, the differences of the RVP values could be evaluated by parametric statistical methods. The Bland–Altman diagram is shown in Fig. 2.

As seen from the diagram, only a single observation was outside the limits of mean ± 2 s by + 0.5 mmHg. According to the confidence interval formula of McAlinden et al. [16],

$$desired\;confidence\;interval\;of\;limits\;of\;agreement=1.96\sqrt[2]{\frac{3{s}^{2}}{n}}$$

Therefore, with *n* = 36 participants, one expects to achieve an accuracy of 0.65 s. The concordance correlation coefficient according to Lin was found to be 0.96 [17] which indicates an almost complete agreement of the measured values of the two methods.

The agreement in tolerability between the IOPstim and CLD was assessed using the McNemar test which was applied only to classes 1 and 2 (due to a non-sufficient sample size in classes 3 and 4).

The difference in tolerability patterns was found statistically significant (*p* = 0.01).

The patient cooperation was also compared using the McNemar test applied to the three categories excellent, good, and fair. As a result, no significant differences in cooperation patterns were found (*p* = 0.134).

## Discussion

Applanation tonometry was accepted by the ophthalmic community because the authors Goldmann and Schmidt [18] had obtained a high correlation coefficient of 0.99 for the relationship between applanation value with intracamerally measured value on 35 human eyes. These results, judged by the state of the art of statistics at the time, are the basis of intraocular pressure measurement to this day. The statistical tools for assessing the agreement between two measurement methods have since been refined [17]. The presentation of the results with a Bland–Altman diagram and the calculation of the concordance correlation coefficient (CCC) according to Lin is recommended [17]. Judged according to these criteria, there is good to almost complete agreement between the methods examined here. With one exception, the differences are within ± 4.6 mmHg of the mean. In comparison, when new tonometers are approved, the values must be within 5 mmHg up and down from the Goldmann value. In our results according to the CCC with a value of 0.96, there is almost complete agreement between the measured values [17].

When new tonometers are approved, their results must not deviate more than 5 mm Hg upwards or downwards from the values of Goldmann applanation tonometry [19]. In clinical tonometric comparison studies with the GAT, the following standard deviations of the differences were found with approved devices, i.e., ICare—5.2 mmg [19], 3.9 mmHg [20], 2.1 mmHg, 2.5 mmHg[21], and 3.0 mmHg [22]; or with Tonopen—3.0 [23], 2.3 mmHg [21], and 5.6 mmHg [19].

The standard deviation of the differences of 1.15 mmHg in our study is thus smaller than that which is considered acceptable for practice in clinical ophthalmology. Therefore, the limits of agreement were estimated to be 0.15 s less accurate than those proposed by McAlinden et al. [16]. According to their recommendation, at least 100 subjects should be measured in order to reach a good accuracy level [15]. However, due to the limited financial and personnel resources, obtaining the ethics approval and examining 64 more glaucoma patients was not feasible. Most importantly, the precision achieved in our study is clinically acceptable for our patient population.

### Different directions of force vectors

In the CLD measurement, the main force vector aims towards the apex of the orbit and may change the pressure conditions in the region in which the ophthalmic vein leaves the orbit. In the IOPstim measurement, however, the main force vector is directed to the medial wall of the orbit. In this tissue region, no veins are present by which the retinal venous blood is drained. In the measurement of the *arterial* blood pressure at the eye, the influence of the measurement method on the pressure conditions in the orbit is avoided by the suction cup method [24]. Hereby, however, the negative pressure difference necessary for the connection of the suction cup to the eye causes an increase of the intraocular pressure. In most cases, this increased IOP is higher than the expected retinal venous pressure. That makes the suction cup unsuitable for the measurement of the RVP. It can be concluded that the IOPstim may be the method of choice with the least systematic error for the measurement of the RVP.

### Comparison with earlier studies

A couple of studies have been conducted by using the CLD [13, 25–32]. The very high concordance correlation coefficient according to Lin of 0.96 makes it possible to compare the results of the mentioned earlier studies with future results obtained by the IOPstim taking into account that the IOPstim values are 1 mm lower than with the CLD method.

### Repeatability agreeability and cooperation

**Fig. 3:**
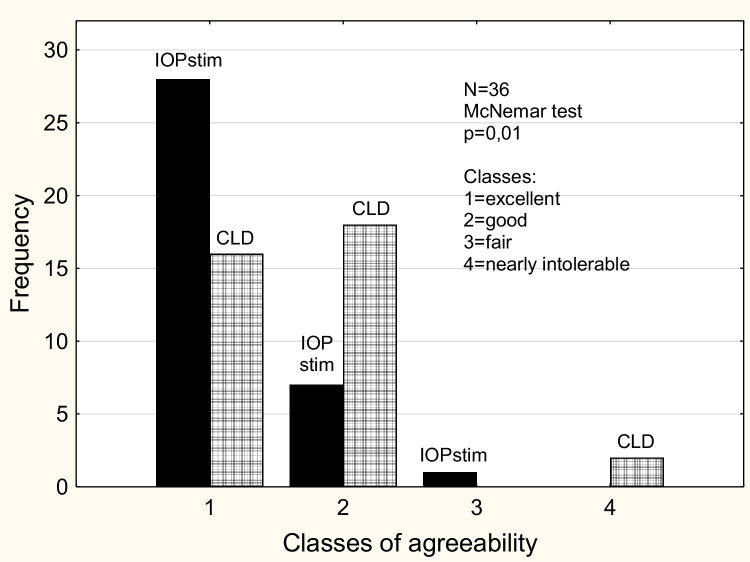
Histogram of the classes of agreeability of the two methods (IOPstim and CLD). The definitions of the classes have been described earlier [8]. For abbreviations, see legends of Fig. 2

The range of the three measurements performed in quick succession (Table 2) is slightly higher in the IOPstim method than in CLD. The high intraclass correlation coefficient, however, can be considered a sign of good reliability of the single values. Figure 3 shows the distribution of the classes of agreeability which was clearly more favorable for the IOPstim method than for the CLD method. Only two patients ranked the CLD method as nearly intolerable. The cooperation pattern of the patients did not differ.

### Comparison with earlier studies

A couple of studies have been conducted by using the CLD [13, 25–32]. The very high concordance correlation coefficient according to Lin of 0.96 makes it possible to compare the results of the mentioned earlier studies with future results obtained by the IOPstim.

### Limitations of the study

A limitation may be the fact that the recognition of the measurement criterion (pulsation or no pulsation) is a subjective process similar to the measurement of the systemic blood pressure by the Riva-Rocci method. The high intraclass correlation coefficient, however, shows that these subjective judgments yield sufficiently reliable results.

## Conclusion

The new IOPstim method for the measurement of the RVP produces sufficiently reliable values of this parameter and is better accepted by the patients as well as the examiner compared to the CLD.

## Declarations

All procedures performed in this study were in accordance with the ethical standards of the institutional ethics committee of the Technical University of Dresden and with the 1964 Helsinki declaration and its later amendments. Informed consent was obtained from all individual participants included in this study. The study was approved by the ethics committee of the Technical University of Dresden (EK 322062019).
